# Quantifying bedside-derived imaging of microcirculatory abnormalities in septic patients: a prospective validation study

**DOI:** 10.1186/cc3809

**Published:** 2005-09-22

**Authors:** E Christiaan Boerma, Keshen R Mathura, Peter HJ van der Voort, Peter E Spronk, Can Ince

**Affiliations:** 1 Department of Physiology, Academic Medical Centre, University of Amsterdam, The Netherlands; 2 Department of Intensive Care, Medical Centre Leeuwarden, The Netherlands; 3 Department of Intensive Care, Gelre Ziekenhuizen Apeldoorn, The Netherlands

## Abstract

**Introduction:**

The introduction of orthogonal polarization spectral (OPS) imaging in clinical research has elucidated new perspectives on the role of microcirculatory flow abnormalities in the pathogenesis of sepsis. Essential to the process of understanding and reproducing these abnormalities is the method of quantification of flow scores.

**Methods:**

In a consensus meeting with collaboraters from six research centres in different fields of experience with microcirculatory OPS imaging, premeditated qualifications for a simple, translucent and reproducible way of flow scoring were defined. Consecutively, a single-centre prospective observational validation study was performed in a group of 12 patients with an abdominal sepsis and a new stoma. Flow images of the microcirculation in vascular beds of the sublingual and stoma region were obtained, processed and analysed in a standardised way. We validated intra-observer and inter-observer reproducibility with kappa cross-tables for both types of microvascular beds.

**Results:**

Agreement and kappa coefficients were >85% and >0.75, respectively, for interrater and intrarater variability in quantification of flow abnormalities during sepsis, in different subsets of microvascular architecture.

**Conclusion:**

Semi-quantitative analysis of microcirculatory flow, as described, provides a reproducible and transparent tool in clinical research to monitor and evaluate the microcirculation during sepsis.

## Introduction

Recent clinical investigations have identified microcirculatory abnormalities as a key component of the pathogenesis of sepsis [1,2]. These new insights have been mainly due to the introduction of orthogonal polarization spectral (OPS) imaging by Slaaf and co-workers [3], which uses green polarized light to observe the microcirculation *in vivo*. Implementing OPS imaging in a hand-held type of tool allowed us to observe the microcirculation of internal human organs for the first time [4,5]. The central role of microcirculatory abnormalities in sepsis was elucidated when OPS imaging was applied in critically ill patients.

Microcirculatory abnormalities were found in septic patients by direct observation of the sublingual microcirculation by means of OPS imaging [6,7], and such abnormalities were found to be predictive in outcome [1].

An important issue in these investigations concerns the method of quantifying the OPS movies of microvascular structures, to identify flow abnormalities associated with sepsis, and evaluate its results. De Backer and co-workers [7,8] introduced a semi-quantitative method, based on the number of perfused vessels crossing three equidistant horizontal and vertical lines. We also developed a score, based on a slightly different principle [6]. Both methods require subjective assessment of flow to identify redistribution between different sized micro vessels, especially the capillaries. Although these methods have proven their worth in practice in identifying the nature of microcirculatory dysfunction in sepsis, neither method has yet been validated in terms of reproducibility. Furthermore, there is a need for a more general method of analysis, applicable to other microvascular structures with different architecture than the usually investigated sublingual vascular bed.

In this study, we present a consensus method of semi-quantitative analysis of OPS imaging that is suitable for quantifying microcirculatory abnormalities in critically ill patients in different subsets of vascular beds: the sublingual region, villi of the small bowel and crypts of the colon. We validated this method for its interrater and intrarater variability and will discuss its potency for future automated analysis by means of software application.

## Materials and methods

### Specifications of the procedure

We called together six collaborative centres involved in clinical microcirculation research in paediatric and adult intensive care units in the Netherlands to come to a consensus about quantification of microcirculatory abnormalities in direct observations obtained by means of OPS imaging. The six centres are involved in OPS studies in various human organ tissues, such as the sublingual region, gut villi, rectal mucosa, skin, conjunctiva, gingival and brain tissue. This was important because we wished to reach a consensus regarding a method that is applicable to the various microcirculatory beds. The aim of the process was to implement a systematic approach to the analysis of OPS derived microcirculatory flow imaging that would allow identification and quantification of microcirculatory abnormalities during critical illness. Preferably, the designed method should be fit to analyse different microvascular structures that have variable vascular anatomy so as to avoid multiple scoring systems for the evaluation of flow imaging in specific organ oriented research. The scoring system should have clear definitions that are easy to teach and have acceptable interrater and intrarater variability. Storage of flow images should be possible at all times and performed in a structured way so that results can be discussed and (re)evaluated. Finally, its application should avoid time-consuming processing and its concept must be suitable for software analysis.

### Definitions

**Figure 1:**
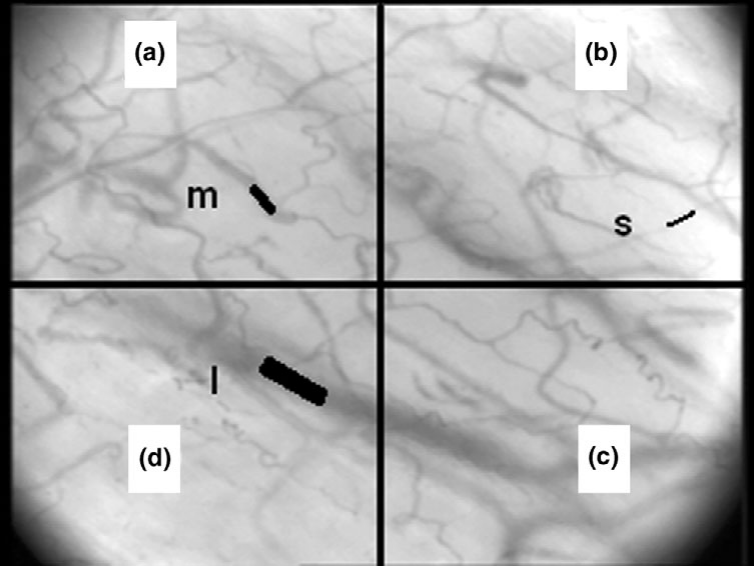
Orthogonal polarization imaging of a microvascular network; the sublingual microvascular architecture. The image is divided in four quadrants (a, b, c and d) with examples of vessel classification: small (s; 10 to 25 μm); medium (m; 26 to 50 μm); large (l; 51 to 100 μm). Objective 5×, on screen 325×.

**Figure 2:**
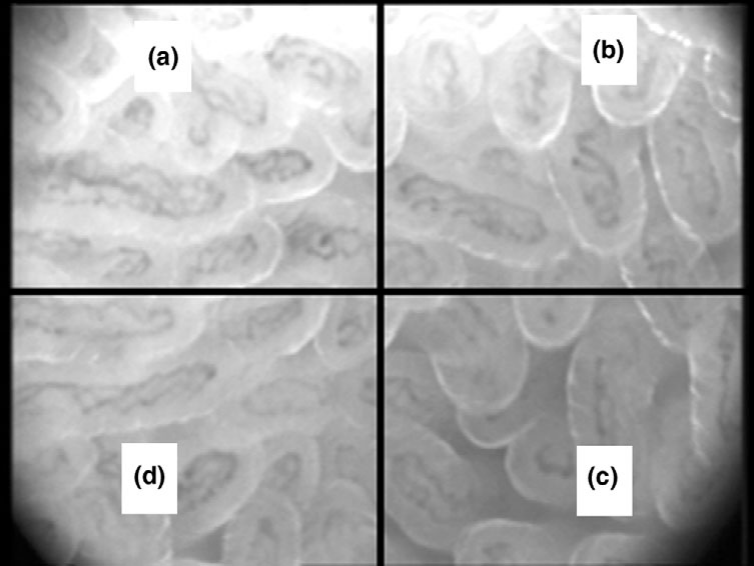
Orthogonal polarization imaging of a repeating vascular structure; the villi of the small intestine. Objective 5×, on screen 325×.

To meet these premeditated qualifications we designed a simple semi-quantitative judgement of microvascular flow, which distinguishes no flow (0), intermittent flow (1), sluggish flow (2) and continuous flow (3). In case a microvascular subunit contains different types of vessels with different diameters (e.g. the sublingual vascular bed), these quantifications of flow can be made per cohort of vessel diameter: small, 10 to 25 μm; medium, 26 to 50 μm; and large, 51 to 100 μm (Figs 1 and 2).

### Imaging technique

The OPS technique, as described in detail elsewhere [9,10], consists of a hand-held device that illuminates an area of interest with polarized light, while imaging the remitted light through a second polarizer (analyser) oriented in a plane precisely orthogonal to the plane of illumination. If a wavelength within the haemoglobin absorption spectrum (e.g. 548 nm) is chosen, red blood cells will appear dark and white blood cells may be visible as refringent bodies. The vessel walls themselves are not visualized directly and their imaging depends, therefore, on the presence of red blood cells.

### Imaging and analysis procedure

After gentle removal of saliva/faeces by an isotonic-saline-drenched gauze, steady images of at least 20 seconds are obtained and stored on digital videotape (SONY video walkman GV-D 1000E^®^), avoiding pressure artefacts. Subsequently, the images are captured in 5 to 10 s representative video clips in avi format (sonyDVgate^®^). Video clips are analysed blindly and at random to prevent coupling between images. Because heterogeneity of flow seems to be an important characteristic of microvascular alterations during sepsis [11], OPS images are obtained from three different regions within the site of interest and each image is divided into four equal quadrants (A,B,C and D). Quantification of flow is scored per quadrant, for each cohort of vessel diameter if applicable. The overall score, called microvascular flow index, is the sum of each quadrant-score divided by the number of quadrants in which the vessel type is visible (Tables 1 and 2).

### Setting and patient selection

To validate the above process of quantification, we performed a single centre prospective observational validation study in a tertiary teaching hospital with a 23 bed mixed intensive care unit. During an eight month period, patients with a new stoma in the course of abdominal sepsis were included. Overt clinical necrosis of the stoma was a contraindication for OPS imaging. This particular model was chosen because a complete spectrum of microvascular flow abnormalities, ranging from no flow (0) to normal flow (3), was expected to be visualized in potentially three different microvascular subsets: the sublingual region, gut villi in an ileostomy and crypts in a colostomy. A local ethical and scientific committee waived the need for informed consent as the observations were considered non-invasive and no interventions were made.

### Statistical analysis

Interrater and intrarater variability was calculated by kappa (κ) cross tables for ordinal variables in Analyse-it^® ^(Analyse-It Software, Leeds, UK) and presented with 95% confidence intervals (CI). The advantage of κ-coefficient calculation, above establishing agreement alone, lies in the fact that the κ-coefficient also takes into account the rule of chance [12,13]. The chance of agreement was estimated to be considerable with such a limited number of ordinal variables. A κ-coefficient >0.6 was considered good [13]. Weighted κ-coefficients (κ_w_) were additionally calculated in order to take into account the level of disagreement, giving weights to disagreement according to the magnitude of the discrepancy [14].

## Results

In an eight month period, 12 patients were included with a new stoma as part of treatment of an abdominal sepsis. OPS imaging was performed both in the sublingual region and in a stoma during the intensive care unit stay on days 1, 3 and 7 after the surgical procedure. In five patients an ileostomy, and in seven patients a colostomy, was constructed. The mean APACHE II score of the included patients was 19.7 (standard deviation ± 7.97) with an observed 45% intensive care unit and hospital mortality. All patients were ventilated.

For assessment of interrater variability, each of two blinded investigators scored the flow in each sample independently. For the sublingual region there were 224 samples available. In 202 (90%) samples there was complete agreement; a scoring difference of -1/+1 was found in 22 (10%) cases (Table 3). The κ-coefficient for interrater variability in the sublingual region was 0.85 (0.79–0.91; Table 4). As agreement in this sample size appeared to be this good, further analysis was done in a reduced sample size (arbitrarily a 50% reduction of all available data was chosen). Stoma flow interrater agreement was complete in 85/96 (89%) cases; a -1/+1 difference occurred in 11/96 (11%) cases (Table 5) with a κ-coefficient for the combined stoma site of 0.84 (95% CI 0.75–0.93;Table 4).

To assess intrarater variability, flow was scored two times independently by the same investigator. For sublingual flow, complete intrarater agreement was found in 86/100 (86%) samples, a -1/+1 difference in 12/100 (12%) and a -2/+2 difference in 2 (2%) cases (Table 6). The intrarater variability κ-coefficient was calculated to be 0.78 (0.67–0.89) for the sublingual region (Table 4). Stoma flow intrarater agreement was complete in 64/72 (89%), a -1/+1 difference occurred in 8/72 (11%) cases (Table 7). The κ-coefficient for intrarater variability for the combined stoma sites was 0.83 (0.71–0.94;Table 4).

## Discussion

We have shown that interrater and intrarater agreement and the κ-coefficient for our method of semi-quantitative analysis of OPS imaging of the microcirculation is high. This appears to be true for different microvascular structures. These results are important because the introduction of OPS flow imaging in the field of clinical research has provided new perspectives, unravelling the complex pathophysiology of microvacular dysfunction during sepsis. For the first time alterations of human microcirculatory flow could be visualized *in vivo *[4,5]. In combination with sublingual capnometry [15,16] or near infrared spectroscopy for measuring microcirculatory haemoglobin saturation [17,18], OPS imaging can be used to investigate the relationship between the microcirculation and metabolic state during sepsis. Persistent microvascular disturbances in the sublingual vascular bed during sepsis are associated with poor outcome, providing a tool for detecting distributive defects in sepsis, which could not achieved by conventional monitoring of systemic hemodynamic- or oxygen-derived variables [1]. Furthermore, therapeutic interventions, such as the use of volume resuscitation, vasopressors and vasodilators [6,19], can be monitored at their potential level of impact, the microcirculation. This promise can only be realised, however, when the obtained images are interpreted uniformly and quantification of microcirculatory flow abnormalities is reproducible.

To compare and evaluate OPS-derived flow imaging, it is essential to quantify the complete spectrum of flow disturbances during sepsis and other shock models. Although direct measurement of red blood cell velocity in a separate vessel is very well feasible, its application does not do justice to the complex microcirculatory flow patterns during sepsis, in which heterogeneity of flow seems to be a key characteristic [11]. It is important, therefore, to quantify a complete flow-pattern in a specific organ site, preferably in more than one location. Hence, the choice not only to derive OPS images from three different locations within the organ site, but also to divide the image itself into four quadrants. The definitions of different flow patterns were kept simple (no flow, 0; intermittent flow, 1; sluggish flow, 2; and continuous flow, 3) to avoid misconstruction. The overall good agreement in the quantification of flow, per group of vessel diameter if applicable, validates its transparency and reproducibility. Important for future implementation of this semi-quantitative flow score in clinical research or even clinical practice, is the fact that disagreement of flow quantification greater than +1/-1 was virtually absent, as expressed by the weighted κ-coefficients, thus eliminating the possibility of interchanging normal flow patterns with clearly pathologic flow patterns.

During sepsis, a standstill, interruption or decrease of red blood cell velocity might not be the only characteristic of microcirculatory flow as hyperdynamic microcirculatory flow patterns have also been observed. Because an increase in red blood cell velocity may also lead to shunting, by means of the inability of haemoglobin to off-load oxygen fast enough to tissues as it passes through the microcirculation [20], it seems important to distinguish normal flow from hyperdynamic flow as well. With the current OPS technique being recorded at 25 frames per second, however, it is not possible to detect these differences in flow adequately. In the future, these limitations might be overcome by a new imaging technique with a considerably better resolution: Sidestream Dark-Field imaging [21]. Under these conditions, a category 4 might be added to the flow variables.

The described type of analysis is especially suited for images derived from non-fixed positions of a hand-held device. Under these circumstances, the exact length of the vessel can not be determined, preventing the exact quantification of red cell velocity and vessel diameter. The highly improved image quality of Sidestream Dark-Field imaging has now made it possible, however, to apply process algorithms much more effectively. To date, we have developed image-processing software designed for vessel identification in vascular images using a process known as segmentation. Velocity is determined semi-automatically after constructing space-time diagrams from the centreline intensity of vessels in subsequent video frames. It allows the user to query length, width and blood velocity of individual vessel segments, thus creating a detailed statistical report containing vascular parameters.

To avoid a complex set of non-comparable quantification systems for individual organ sites, the presented way of semi-quantitative analyses was not only designed for the evaluation of the behaviour of microcirculatory networks such as the sublingual region and the brain [6], but also for repeating vascular structures like those in the small intestine (villi), colon (crypts), rectum (crypts), liver (sinuses) and gingival tissue [22]. Intrarater and interrater agreement and κ-coefficient for semi-quantitative flow analysis in stomas of the small intestine and colon were as good as those for sublingual microcirculatory structures. This way of flow quantification seems, therefore, potentially applicable to the analysis of OPS imaging in many more microvascular structures not yet described in the literature.

## Conclusion

Semi-quantitative analysis of OPS derived flow imaging, as described, has a good intrarater and interrater reproducibility for the evaluation of microcirculatory flow patterns during sepsis, both for microcirculatory networks and for repeating microvascular structures. It provides a transparent and clinically applicable non-invasive tool to monitor and evaluate the microcirculation at the bedside.

## Key messages

• 	Semi-quantitative analysis of OPS derived flow imaging, as presented, has good interrater and intrarater reproducibility.

• 	The described method of analysis is applicable both for microcirculatory networks and for repeating microvascular structures.

• 	It provides a transparent, easy to use, clinical, non-invasive tool to monitor and evaluate the microcirculation at the bedside.

## Abbreviations

CI = confidence interval; OPS = orthogonal polarization imaging.

## Competing interests

The author(s) declare that they have no competing interests.

## Authors' contributions

CB contributed to the design of the study, performed OPS imaging and analysis and drafted the manuscript. KM coordinated the consensus conference, provided technical support and revised the manuscript. PV performed statistical analysis and revised the manuscript critically. PS contributed to the design of OPS imaging analysis and revised the manuscript. CI conceived the study, participated in its design and coordination and helped to draft the manuscript. All authors read and approved the final manuscript.

## Figures and Tables

**Table 1:** Example of microvascular flow index calculation for a (sublingual) microvascular network

Flow	Quadrant A	Quadrant B	Quadrant C	Quadrant D	MFI
Small	2	3	3	2	10/4 = 2.5
Medium	1	3	3	3	10/4 = 2.5
Large	-	3	3	-	6/2 = 3

MFI, microvascular flow index.

**Table 2:** Example of microvascular flow index calculation for a repeating microvascular structure (gut villi)

	Quadrant A	Quadrant B	Quadrant C	Quadrant D	MFI
Flow villi	2	3	3	2	10/4 = 2.5

MFI, Microvascular flow index

**Table 3:** Inter-observer agreement for flow score in the sublingual region

	Observer 1				
Observer 2	Flow 0	Flow 1	Flow 2	Flow 3	

Flow 0	16	2	0	0	
Flow 1	2	22	3	0	
Flow 2	0	4	65	8	
Flow 3	0	0	3	99	
				Total	224

**Table 4:** Statistical data for semi-quantitative flow scoring in the sublingual region and in combined stoma sites

Reliability	Agreement	Chance	Kappa^a^	κ_w_
Sublingual				
Interrater	0.90	0.35	0.85 (0.79–0.91)	0.90
Intrarater	0.86	0.37	0.78 (0.67–0.89)	0.81
Stoma				
Interrater	0.89	0.28	0.84 (0.75–0.93)	0.89
Intrarater	0.89	0.36	0.83 (0.71–0.94)	0.89

^a^Kappa plus 95% confidence intervals between brackets; κ_w _= weighted kappa coefficient.

**Table 5:** Inter-observer agreement for flow score in the combined stoma sites

	Observer 1				
Observer 2	Flow 0	Flow 1	Flow 2	Flow 3	

Flow 0	9	3	0	0	
Flow 1	0	21	1	0	
Flow 2	0	6	29	1	
Flow 3	0	0	0	26	
				Total	96

**Table 6:** Intra-observer agreement for flow score in the sublingual region

	Observer 1				
Observer 2	Flow 0	Flow 1	Flow 2	Flow 3	

Flow 0	4	0	0	0	
Flow 1	0	10	2	2	
Flow 2	0	1	30	7	
Flow 3	0	0	2	42	
				Total	100

**Table 7:** Intra-observer agreement for flow score in the combined stoma sites

	Observer 1				
Observer 2	Flow 0	Flow 1	Flow 2	Flow 3	

Flow 0	9	0	0	0	
Flow 1	1	3	1	0	
Flow 2	0	1	32	1	
Flow 3	0	0	4	20	
				Total	72

^a^Kappa plus 95% confidence intervals between brackets; κ_w _= weighted kappa coefficient.
